# Core components of an anti-racist approach among health professions educators: an integrative review

**DOI:** 10.5116/ijme.64e9.b6b4

**Published:** 2023-09-15

**Authors:** Amelie Blanchet Garneau, Patrick Lavoie, Vanessa Sit, Catherine Laurent Sédillot

**Affiliations:** 1 Faculty of Nursing, Université de Montréal, Canada; 2 Department of Anthropology, Cégep Édouard-Montpetit, Canada

**Keywords:** Racism, anti-racist pedagogy, health professions education, integrative review

## Abstract

**Objectives:**

This integrative literature review aimed to identify the core elements of an anti-racist approach among health professions educators.

**Methods:**

We searched five databases CINAHL (EBSCOhost), ERIC (ProQuest Dissertations & Thesis Global), EMBASE (Ovid), MEDLINE (Ovid), and Web of Science (Social Sciences Citation Index, Citation Index Expanded) in March 2021. The search strategy combined concepts related to anti-racist pedagogies in the context of health professions education by educators in any capacity. From 1,755 results, we selected 249 manuscripts published in English or French between 2008 and 2021 based on titles and abstracts. After reviewing the full texts, we selected the 48 most relevant sources. We extracted data regarding knowledge, skills, and attitudes in reference to anti-racist approaches or surrogate terms. Within each category, we grouped similar data using a conceptual map.

**Results:**

Analysis of the selected sources revealed that, for health professions educators, engaging in an anti-racist pedagogical approach requires more than incorporating racialized perspectives and content into the classroom. It rather rests on three interrelated components: developing a critical understanding of power relationships, moving toward a critical consciousness, and taking action at individual and organizational levels.

**Conclusions:**

This review sheds light on knowledge, attitudes and skills that educators must deploy to adopt an anti-racist approach competently. This approach is a learned, intentional, and strategic effort in which health professions educators incorporate anti-racism into their teaching and apply anti-racist values to their various spheres of influence. This ongoing process strives for institutional and structural changes and requires whole-system actions.

## Introduction

Structural racism is a core determinant of health, social, political, and economic inequities racialized and marginalized populations face.^1^ Many medical and nursing associations recommend decolonizing health professions education as one way to tackle racism and discrimination in healthcare.^2^^-^^4^ Decolonization means that health professions educators must learn to adopt an anti-racist approach to open, support, and deepen dialogue with students about issues related to racism^5^ and go beyond teaching about culturally specific knowledge and practices^6^^,^^7^ that risk perpetuating stereotypes and “othering” of racialized populations. Anti-racist pedagogy is a critical theory paradigm that explains and counteracts the persistence and impact of racism by using praxis to promote social justice for creating a democratic society.^8^ It aims to identify, question, and transform aspects of a system that maintain and reproduce inequities and conditions of racism, power, and privilege.^9^ However, many health professions educators are reluctant and actively avoid doing so.^10^^,^^11^ This discomfort is often attributed to a lack of knowledge or appropriate training.^12^ The requirements for adopting an anti-racist approach are manifold and complex. Because of the deeply entrenched nature of colonization in current educational and health systems, institutions are often poorly equipped to support educators in adopting an anti-racist pedagogy.

Before addressing issues related to racism in health profession education, it is imperative to define and build health professions educators’ capacity to adopt anti-racist approaches in their practice.^13^ From a constructivist perspective, teaching with an anti-racist approach is a competency that requires educators to mobilize and combine a variety of resources (i.e., knowledge, skills, and attitudes) to effectively respond to various pedagogical situations as they unfold in context.^14^

Although a large body of literature emphasizes the importance of training in anti-racist pedagogy^3^^,^^15^^,^^16^ a much smaller body of literature focuses on the resources educators need to implement such an approach in practice.^17^ Few studies have described educators' experience addressing healthcare discrimination issues.^10^^, ^^13^ These studies provide insight into certain characteristics of educators’ competence specific to a particular context. Examples of strategies to support educators include workshops to explore unconscious biases,^18^ training on individual and systemic discrimination issues and their effects on health^19^ and reflection on their attitudes toward racism.^20^ Such strategies mostly prepare educators for the content to be taught without identifying the resources needed or providing tools and methods to implement an anti-racist approach.

This integrative review aimed to identify core components of an anti-racist approach among health professions educators. The question that guided this effort was: “What knowledge, attitude, and skills does an educator need to teach with an anti-racist approach?”

## Methods

An integrative literature review is a review method that summarize theoretical and empirical literature to provide a comprehensive understanding of a healthcare problem or phenomenon.^21^ We followed the updated integrative review methodology developed by Whittemore and Knafl^21^ and Toronto and Remington^22^ guide to conducting integrative reviews to guide problem formulation, literature search, data evaluation, data analysis, and presentation of the results, as presented in the following sections.

### Search strategy

We considered sources published in English or French without year restriction. We considered empirical studies, theoretical literature, doctoral dissertation, reports, and reviews published in peer-reviewed journals or revised by a scientific committee.

The target population for this review was educators, including future teachers, university professors, internship supervisors, mentors, preceptors, and tutors. Because some authors point out that the needs of educators from racialized minorities are poorly considered,^23^ we ensured inclusion of texts specifically addressing racialized faculty development and issues related to anti-racist teaching for racialized faculty.^24^^-^^26^

Concepts of interest were related to critical pedagogies, including anti-racist pedagogy, decolonizing pedagogy, anti-oppressive pedagogy, and anti-discriminatory pedagogy because work on decolonization, indigenization, and cultural safety of teaching and teacher education often refers to anti-racist approaches.^4^^,^^27^^-^^32^ For the context, we were interested in health professions education, including initial and continuing education and professional development.

The search strategy combined concepts related to critical, anti-racist, decolonizing, anti-oppressive, and anti-discriminatory pedagogies in the context of health professions education (pre- or post-licensure) by current or future educators. We conducted comprehensive searches in five databases: CINAHL (EBSCOhost), Education Resources Information Center (ERIC, ProQuest Dissertations & Thesis Global), EMBASE (Ovid), MEDLINE (Ovid), and Web of Science (Social Sciences Citation Index, Citation Index Expanded) in March 2021. From 1,755 results, 249 manuscripts published in English or French between 2008 and 2021 were first independently selected by two authors (CLS, VS, ABG). The reference lists of identified articles were hand-searched for additional records. Records were exported into Endnote, and duplicates were removed. Two authors independently assessed titles, abstracts, and then the full texts of potentially eligible records. At each stage, disagreements were resolved by discussion or involvement of a third author. We selected a total of 48 references, which were included based on an evaluation of methodological or theoretical rigor and relevance to this review’s purpose.

### Data extraction and analysis

We extracted data from primary sources regarding knowledge, skills, and attitudes in reference to anti-racist approaches (or surrogate terms). Within each category, we grouped similar data using a conceptual map. The conceptual map helped display all the coded data from each source by category and compare them iteratively. We reviewed primary sources as we conceptualized data at a higher level of abstraction to verify that the renewed conceptualization was congruent with primary sources and ensure accuracy and confirmability. As a result, we developed three core components of an anti-racist approach among health professions educators related to knowledge, attitudes and skills: developing a critical understanding of power relationships, moving towards a critical consciousness, and taking action at individual and organizational levels.

## Results

The 48 selected references include 40 articles (n=16, 33% theoretical and n=24, 50% empirical), 3 doctoral dissertations (6%), 1 master thesis (2%), 2 reports by non-governmental agencies (4%), and 2 literature reviews (4%). They are mostly from the United States (n = 26, 54%), but also from Canada (n= 17, 35%), Australia (n=2, 4%), Brazil (n=1, 2%), South Africa (n=1, 2%) and the United Kingdom (n=1, 2%). Their authors come from a variety of disciplines, but mainly from the science of education and the philosophy of education (n=20, 42%), the health professions (medicine, nursing, midwifery; n=19, 39%), and social work (n=4,8 %). The purposes of the selected sources were to present 1) the design, implementation, or evaluation of faculty and professional development training and programs (n=17, 35%), 2) the design, implementation, or evaluation of the successes and barriers of anti-racist curricula, teaching approaches, or programs (n=16, 33%), and 3) the perceptions and experiences of educators about racism, anti-racism, or using an anti-racist approach (n=5, 10%).

Analysis of the selected sources revealed that, for health professions educators, engaging in an anti-racist pedagogical approach requires more than merely incorporating Indigenous or racialized perspectives into the classroom. It rather rests on three interrelated components: developing a critical understanding of power relationships, moving towards a critical consciousness, and taking action at individual and organizational levels.

### Knowledge: Developing a critical understanding of power relationships

To engage in genuine dialogue about race and racism with students, health professions educators must understand the complexity of racism and its various forms (individual, internalized, institutionalized, systemic) within broader sociopolitical and historical contexts inseparable from colonialism.^5^^, ^^10^^, ^^33^^, ^^34^ This knowledge is required to develop a critical understanding of the power relationships they are part of and participate in. However, many educators still primarily view the concepts of race as a biological category and “racism” in terms of problematic individual attitudes; their understanding of systemic racism remains embryonic.^10^ Therefore, they must understand the intersectionality of systems of oppression (race, class, gender, and sexuality) and their effects on the construction of inequities and the objective conditions of people's lives.^17^^, ^^23^ This deeper understanding requires mastery of concepts such as social justice, whiteness, white privilege, white supremacy, race, unconscious bias, microaggression, prejudice, and stereotyping.^17^^,^^35^^-^^37^ Authors suggest teaching white educators about the historical, political, and social construction of whiteness to help them understand this aspect of their identity and their emotional engagement with it.^17^^, ^^36^

Anti-racist education is necessarily critical of hegemonic and Eurocentric knowledge (especially so-called "objective and rational" knowledge) and its supposed universality.^17^^, ^^23^^, ^^25^^, ^^33^ It opposes the hierarchization of knowledge and reveals that all knowledge is necessarily situated, constructed, partial, and shot through with power relations.^23^^, ^^25^^, ^^26^ For this reason, educators must develop a critical knowledge of the historical, political, and economic context in which their discipline has developed.^17^ This includes understanding the factors that maintain the underrepresentation of racialized students and educators and the various barriers to their success.^17^ In Canada, for example, educators must know the history of colonization, occupation of the territory, laws, treaties, and attempts to acculturate Indigenous Peoples, mainly through the residential school system, and the persisting consequences for Indigenous Peoples today.^31^^,^^38^^,^^39^ In the United States, it is about knowing the history of segregation and the civil rights movement,^10^^,^^17^ but also the recent backlash against the movement (e.g., anti-immigration policy, social net reforms at the expense of racialized populations.^17^

### Attitudes: Moving toward a critical consciousness

Becoming aware that a set of hegemonic social and historical practices, norms, ideologies, systems of power, and privileges participate in the reproduction of inequities is central to the development of critical consciousness among educators.^5^^, ^^10^^, ^^25^^-^^27^^, ^^38^ It means for educators to become conscious of their racial identity, worldview, and social position to understand: 1) how these have been and continue to be a source of privilege or internalized oppression^40^ and 2) how these structure their classroom interactions, pedagogical approaches, content, and assessments^5^^,^^27^^,^^36^^,^^41^^,^The need for white educators to engage in critical self-reflection is the subject of significant literature.^42^^-^^44^

Anti-racist pedagogy is based on values of power-sharing and democratic decision-making, fairness, responsibility to others, openness, and transparency. "It is not about imposing anti-racist values on others but practicing those values [our]selves."^17^ Embodying these values involves educators rejecting the elitism of academia, having the humility to recognize what one can learn from others, and engaging in an ongoing learning process.^17^ It also involves acknowledging that learners come with their backgrounds and rich experiences.^18^^,^^40^^, ^^41^ Moreover, this humility translates into recognizing the value of sharing with racialized and non-racialized colleagues to address difficult situations^35^^,^^45^ and reflect on one’s practices and assumptions.^27^^, ^^46^

### Skills: Taking action at individual and organizational levels

For health profession educators, implementing an anti-racist approach implies learning to create a safe learning environment, value non-hegemonic knowledge diversity, deal with uncomfortable and confronting situations, and actively engage in decolonizing their institutional settings and communities. Each educator’s implementation of an anti-racist approach is unique. It must consider the specific sociodemographic, socioeconomic, cultural, and racial makeup of the classrooms, institutions, and communities in which it is deployed.

Anti-racist educators learn to create classroom climates that allow students to feel welcomed and safe to address emotionally charged topics such as racism and colonialism.^5^^, ^^25^^, ^^27^ Establishing a climate conducive to critical questioning and active listening requires acknowledging the dialectical tension between their desire to learn with students and the authority and power inherent in the educator's role.^23^ Educators may engage in anti-authoritarian, student-centred educational practices to foster a sense of community and non-competitiveness in the classroom.^17^^, ^^31^

Anti-racist educators learn to cultivate spaces conducive to the emergence of non-hegemonic knowledge and develop pedagogical content that values Indigenous epistemologies and cultures^25^^, ^^27^^, ^^32^^, ^^38^ as well as the forms of knowledge and experiences of minority and racialized groups.^17^^,^^25^^,^^42^ As Kishimoto^17^ points out, this does not mean reifying binary oppositions (white/Black; Indigenous/non-Indigenous) and essentializing the experiences of group members but exposing the diversity of lived experiences. For Boutouchent,^38^ it is therefore crucial that programs only prescribe learning objectives and allow educators to choose the best ways to achieve them, leaving room for creativity.

Anti-racist educators also learn to deploy a "pedagogy of discomfort"^5^ which is to feel comfortable in situations of conflict and confrontation that necessarily emerge from discussing racism.^5^^,^^23^^,^^26^^,^^47^ This includes developing their communication skills to facilitate difficult discussions^10^ and navigate through silences and unspoken words.^10^^,^^23^ Acosta and Ackerman-Barger^5^ suggest strategies such as the "courageous conversation" theorized by Singleton.^48^ It is also essential to develop their ability to be vulnerable in the classroom and share their stories and experiences in ways that demonstrate their own learning pathway.^47^

Beyond the classroom, anti-racist educators commit to equity, inclusion, and decolonization in their institutions and communities. They commit to retaining racialized educators^17^ to increase the excellence of the education offered to students. Partnering with racialized colleagues to teach, research, or serve on committees, or simply talking informally are ways to participate in their retention.^36^ Anti-racism also means nurturing relationships, responsibility, solidarity, and commitment to colleagues and students.^49^ Incorporating power-sharing and democratic decision-making, accountability mechanism, openness, and transparency also means becoming more involved with families, students, and the communities in which educators work,^32^ gaining their trust, and building relationships and partnerships that benefit them. Working on various projects with community members, elders, and other knowledge keepers, while being aware that these resource persons are over-solicited also goes in this direction.38 These partnerships must be based on reciprocity, accountability, transparency^17^ and reconciliation.^50^

## Discussion

The results of this integrative review capture interrelated knowledge, attitudes and skills needed for educators to implement an anti-racist approach to health professionals’ education. The three core components of an anti-racist approach, namely developing a critical understanding of power relationships, moving toward critical consciousness, and taking action at individual and organizational levels, are explicitly located within a critical theoretical framework. The entrenched nature of colonization and its effects on institutions and educators’ practices brings the need to rethink deeply teaching practices and institutions, which are too often complicit in reproducing racism, colonialism, and other systems of oppression.

Based on the core components of an anti-racist approach resulting from this review, several challenges to its implementation in health professions education are to be expected. The multiculturalist and individualist perspectives in which the health professions are rooted^51^ are among them. Indeed, health professions education is still dominated by multicultural, intercultural, or cultural competence approaches.^52^ These approaches convey an unpoliticized vision of "diversity," celebrated for its richness. They fail to problematize issues of race and racism and do not allow learners to develop a deeper understanding of power relations and oppression at the systemic level. Like the color-blindness and equality discourses in healthcare, they invisibilize racism and promote the status quo.^5^^,^^17^^,^^40^^,^^51^ An anti-racist approach shifts learners’ attention from presumed differences between racialized populations’ cultures and Eurocentric dominant cultures as the source of problems. Instead, education is directed toward addressing the root causes of inequitable power relations and health outcomes affecting racialized populations, the health and social impacts of racism, and strategies for creating more equitable systems of care for racialized populations in service delivery contexts.^53^^, ^^54^

In the reviewed work, analyses are primarily at a systemic level and indicate a need to move from an individual-level analysis to critical theoretical groundwork. In order to develop a critical understanding of power relationships, a critical consciousness, and take action at individual and organizational levels, educators need to focus on a structural analysis of health professions education and advocate for an extensive transformation of programs. Thus, implementing an anti-racist approach in health professions education requires structural changes. However, anti-racist content and racialized populations’ perspectives are still mostly integrated by adding one or a few courses or the optional offering of professional development workshops of a few hours or days.^23^^,^^36^^,^^49^ In line with critical theoretical perspectives, the core components of an anti-racist approach among health professions educators highlight the need to deploy an anti-racist approach beyond classrooms with the support of institutions. Health professionals have a leadership role to play in understanding and counteracting the impacts of colonialism in their education and practice. Waite and Nardi^55^ propose strategies to decolonize the nursing profession, including confronting "white silence," critiquing knowledge development and supporting change in the curriculum. However, a lack of institutional support is a significant challenge to ensuring anti-racist teaching, especially in a context where many lecturers or associate professors are assigned the responsibility of teaching diversity and equity courses without adequate doctoral training or professional support.^47^ A change in institutional culture can be supported by incorporating mandatory implicit bias training for all faculty, staff, and students.^56^ Several authors also see an institutional commitment to equity, anti-racism, and decolonization as requiring sustained investments (organizational and financial) in the professional development of their faculty^36^^,^^42^ and clear institutional policies and practices for hiring and retaining racialized faculty.^31^^, ^^38^ Thus, beyond individual educators becoming anti-racist educators, institutional change, accountability mechanisms, and whole-systems actions are required to provide anti-racist education to health professionals.^36^^, ^^42^^, ^^47^^, ^^53^

### Implications for medical education

In medical education, along with train-the-trainer tools and training opportunities, there is a need to develop tools to implement an anti-racist pedagogical approach in health professions education within classrooms and beyond them. The core components of an anti-racist approach in health professions education shed light on the multiple requirements for adopting an anti-racist approach. Both educators and faculties wishing to move away from culture-based teaching, which is still widely used in the training of health professionals and has little effect on the transformation of discriminatory practices,^29^ will benefit from it. Such conceptualization can also serve as a basis for developing professional development tools (e.g., portfolio, continuing education plan) and assessing the quality of teaching. The development of this knowledge can therefore guide the practice and professional development of health science educators so that they are better prepared to develop and implement a curriculum that challenges racism and discrimination in health care for future health professionals. Since teaching from an anti-racist perspective involves examining various forms of discrimination and the implications of relative privilege and systemic disadvantage, the results may be extended to other professional training such as social work and education. Along with professional development tools to implement an anti-racist approach in medical education, further research is needed to assess the impacts on the development of educators' knowledge, attitudes and skills, and the transformation of educators’ practices and educational institutions.

### Strengths and limitations

The inclusion of diverse data sources is a strength of this review because it enhances a holistic understanding of an anti-racist approach in health profession education. We also employed techniques of qualitative research to this review process, which has the potential to reduce bias and error. In addition, we rigorously followed Whitemore and Knafl’s^21^ and Toronto and Remington’s^22^ guidelines to strengthen this review's process and outcomes. However, three out of four authors of this integrative review identify as white settlers. Although some are trained anti-racist educators or have participated in cultural safety training grounded in critical perspectives, the influence of the Euro-Canadian dominant perspective has affected the analysis and interpretation of the results of this review.

## Conclusion

This integrative literature review aimed to identify core components of an anti-racist approach among health profession educators. To teach with an anti-racist approach, educators need to develop a critical understanding of power relationships (knowledge), move toward critical consciousness (attitudes), and take action at individual and organizational levels (skills).

An anti-racist pedagogy is not a prescribed method that can simply be applied to our teaching, nor does it end with incorporating racial content into courses. An anti-racist pedagogy is a learned, intentional, and strategic effort in which health professions educators incorporate anti-racism into their teaching and apply anti-racist values to their various spheres of influence. This ongoing process strives for institutional change and requires the collaboration of educators across disciplines, the support of organizations, policy directives, accountability mechanisms, and whole-system actions.

### Conflict of Interest

The authors declare that they have no conflict of interest.
